# Multimodal imaging findings of primary renal well-differentiated neuroendocrine tumors (carcinoid): a case report and literature review

**DOI:** 10.3389/fonc.2025.1597024

**Published:** 2026-01-12

**Authors:** Bangcheng Wei, Yuge Chen, Yueqin Chen, Shujun Zhang

**Affiliations:** 1 Clinical Medical College of Jining Medical University, Jining, Shandong, China; 2 Department of Radiology, Affiliated Hospital of Jining Medical University, Jining, Shandong, China

**Keywords:** primary renal well-differentiated neuroendocrine tumors, carcinoid, multimodal imaging, immunohistochemistry, case report

## Abstract

Primary renal well-differentiated neuroendocrine tumors (NETs) are rare. Due to their non-specific nature, the detection and monitoring of NETs remain highly challenging. Here, we report a case of a 45-year-old man who was admitted after an incidental finding of a space-occupying lesion in the kidney during a routine physical examination. The patient underwent CT and MRI multimodal imaging, followed by pathological and immunohistochemical analysis after surgery, leading to a diagnosis of well-differentiated renal NET. He was subsequently followed up for a long period. A review of the literature, in conjunction with this case, revealed that these tumors present with non-specific clinical and imaging features. Diagnosis primarily relies on pathological and immunohistochemical evaluation. Well-differentiated renal neuroendocrine tumors are associated with low malignant potential and a favorable prognosis. Surgical resection is the treatment of choice, and long-term follow-up is essential to monitor the patient’s condition. By combining this case with existing literature, we aim to provide valuable insights for clinicians in the diagnosis and management of renal NETs.

## 1 Introduction

Neuroendocrine tumors (NETs) are a group of heterogeneous tumors that originate from peptidergic neurons and neuroendocrine cells. The peak incidence of NETs occurs between the ages of 50 and 70, and they can arise in many organs and tissues throughout the body. The most common primary sites are the gastrointestinal tract (62-67%) and the lungs (22-27%). Between 12% and 22% of patients present with metastases at the time of diagnosis. Primary renal neuroendocrine tumors (PRNETs) are rare, accounting for less than 1% of malignant renal epithelial cell tumors (1–3). Currently, the literature primarily consists of case reports and small sample studies. This paper presents the diagnosis and treatment of a 45-year-old male patient with renal NETs and reviews the clinical features, imaging characteristics, pathological findings, treatment options, and prognosis of the disease based on a comprehensive literature review.

## 2 Case report

**Figure 1:**
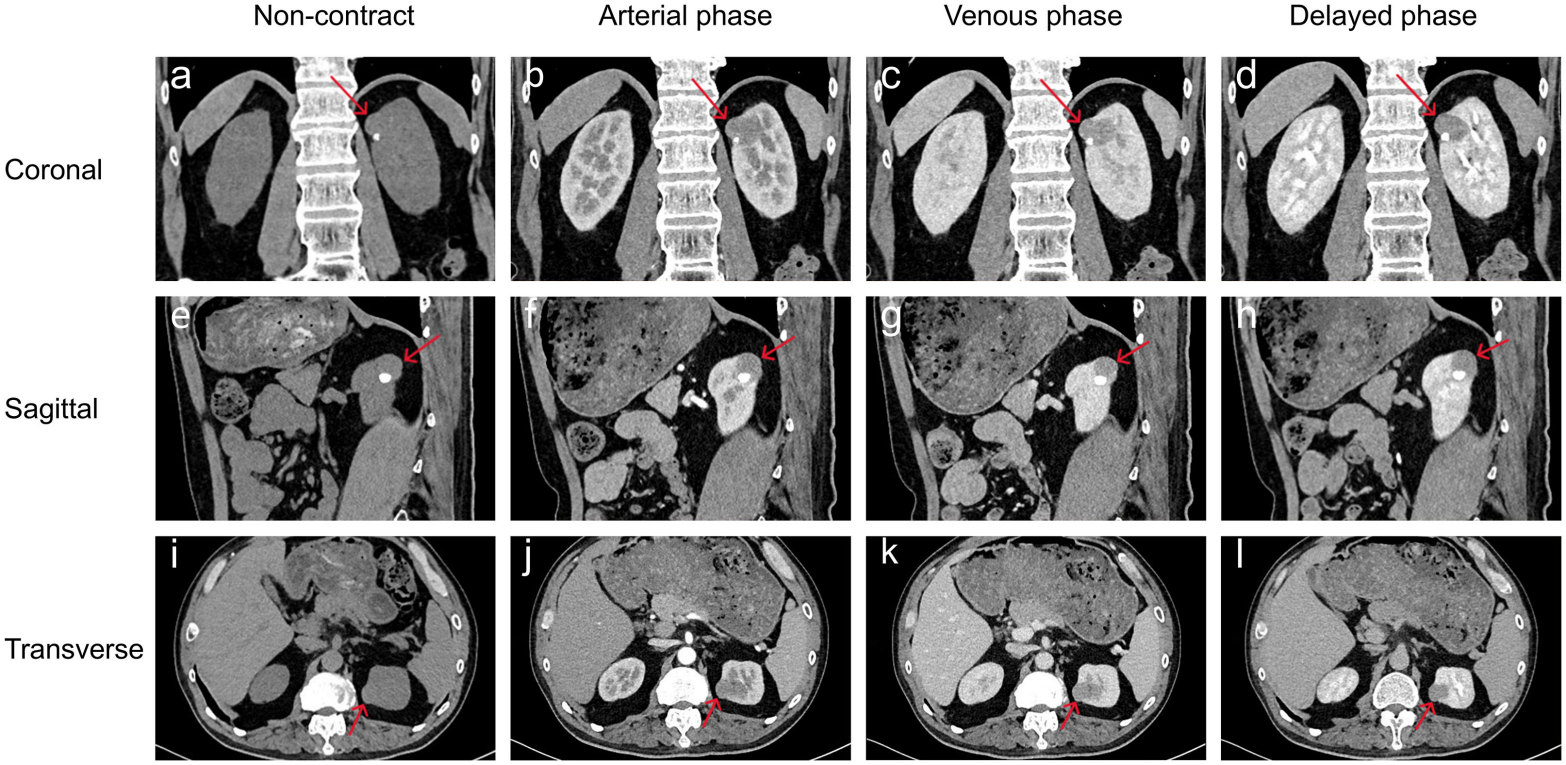
Preoperative renal CT plain scan and enhanced scan. **(a-l)** Multiple nodular iso-low density shadows in the left kidney, locally protruding from the outline of the kidney, and patchy calcification shadow in the left renal parenchyma. Three-phase dynamic contrast-enhanced scans revealed well-defined lesion boundaries, with mild inhomogeneous enhancement in the medial upper pole lesions of the left kidney and the rest showed no enhancement.

**Figure 2:**
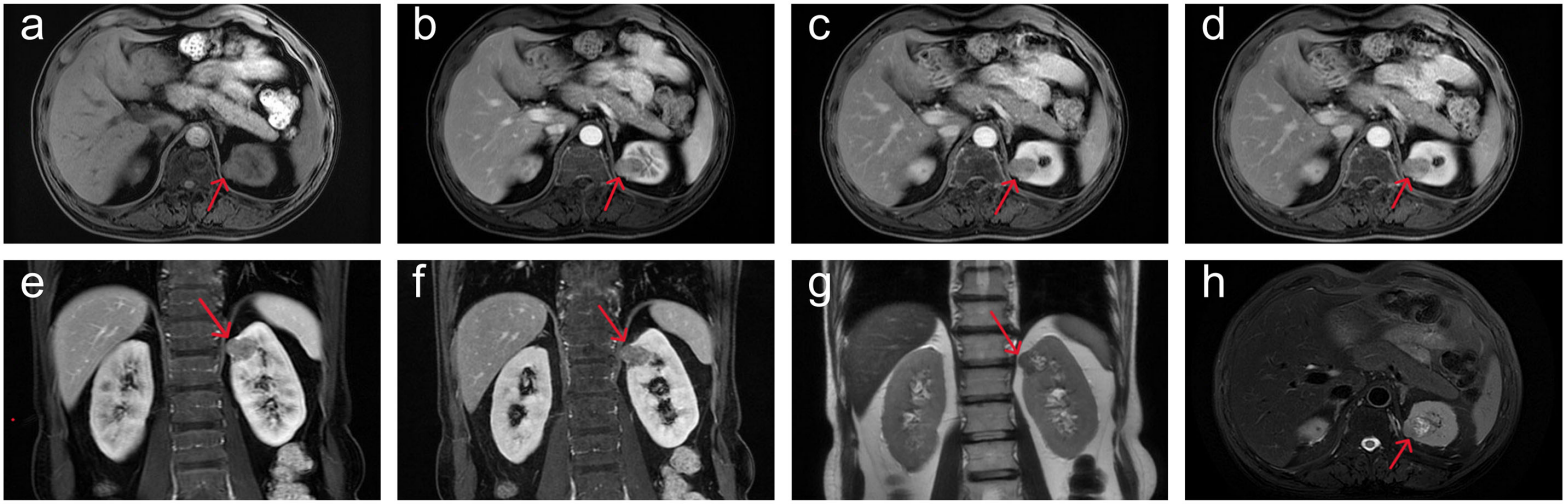
Preoperative renal MRI showed round-like abnormal signal in the upper medial pole of the left kidney. **(a-f)** On contrast-enhanced scan (plain scan, arterial phase, venous phase and delayed phase), the lesions showed mild inhomogeneous enhancement and iso-signal on T1WI. **(g, h)** T2WI showed iso-high mixed signal.

**Figure 3:**
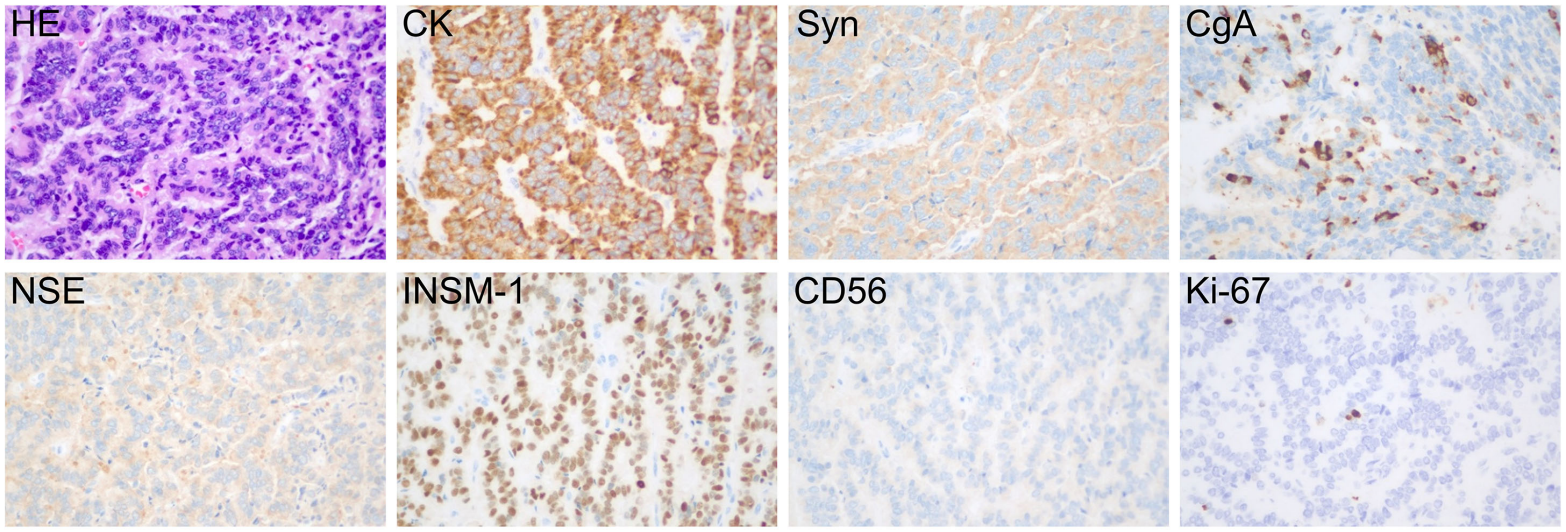
Pathological examination after surgery. The tumor cells were arranged in cords or ribbons, with dichromatic cytoplasm and fine granular or vacuolated nuclear chromatin. Necrosis was not seen, and mitotic activity was rare. Tumor tissue: tumor cells CK (+), Syn (+), CgA (+), NSE (+), INSM-1 (+), CD56 (-), Ki-67 (+, 2-3%). Magnifications ×400.

**Figure 4:**
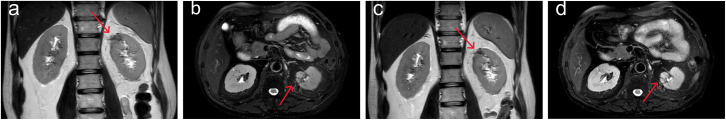
Postoperative MRI Images. **(a, b)** (T2WI): at the 3rd month follow-up, MRI of the lower abdomen showed that after partial nephrectomy, the left kidney was partially absent and irregular, with striped T2WI high signal **(c, d)** (T2WI): at the 7th month follow-up, MRI of the lower abdomen showed that after partial nephrectomy, the left kidney was partially absent and irregular, with striped T2WI high signal.

This study follows the CARE guidelines (4). According to local and national regulations, ethical approval is not required for this study. The patient provided written informed consent, agreeing to receive treatment and to the publication of his medical details, including any accompanying images. A 45-year-old male was diagnosed with a left kidney mass and right kidney stones during a routine physical examination. The patient typically experiences low back pain but does not present with macroscopic hematuria, frequent urination, urgency, dysuria, or painful urination. Physical examination revealed percussion pain in the right renal area but no tenderness in the bilateral ureteral regions, no suprapubic bladder filling, and no tenderness upon palpation. Blood tests, including routine blood work, blood biochemistry, coagulation indices, and inflammation markers, showed no abnormalities. Imaging data: (1)Renal computed tomography (CT): multiple nodular iso- to low-density shadows were observed in the left kidney, with some lesions locally protruding from the kidney’s outline. Patchy calcifications were noted in the left renal parenchyma (**Figures 1a, e, i**). Three-phase dynamic contrast-enhanced scans revealed well-defined lesion boundaries, with mild inhomogeneous enhancement in the medial upper pole lesions of the left kidney and the rest showed no enhancement (**Figures 1b-d, f-h, j-l**). (2)Renal magnetic resonance imaging (MRI): The round-like lesions in the upper pole of the left kidney appeared iso-signal on T1-weighted images (T1WI), iso-high mixed signal on T2-weighted images (T2WI), with a nodular low signal intensity at the edges. These lesions exhibited high signal intensity on diffusion-weighted imaging (DWI), accompanied by a decreased apparent diffusion coefficient (ADC) value, showing a clear boundary. The measured dimensions of the lesion were approximately 27 mm (left to right), 22 mm (anterior to posterior), and 24 mm (superior to inferior). On contrast-enhanced scans, the lesions demonstrated mild inhomogeneous enhancement (**Figures 2a-f**). After admission, preoperative examinations confirmed the diagnosis of a left kidney tumor. After excluding contraindications, laparoscopic partial nephrectomy and perirenal adhesion release were performed. During surgery, the perirenal fascia was incised, the perirenal fat was separated, and the left kidney was exposed. The tumor was excised along with a portion of the kidney tissue. Gross examination revealed a specimen consisting of kidney tissue and surrounding adipose, measuring approximately 4.5×4×2 cm. The cut surface appeared gray-white and gray-red, with a tough texture and areas of calcification. Immunohistochemical analysis (**Figure 3**) showed positive staining for cytokeratin, synaptophysin, chromogranin A, neuron-specific enolase, and insulin-related protein 1, while CD56 was negative and Ki-67 was 2-3%. The pathological diagnosis was renal neuroendocrine tumor (NET, G1, carcinoid) with ossification and negative incisal margins. At the 3-month follow-up, MRI of the lower abdomen showed partial absence and irregularity of the left kidney with striped T1WI low signal and T2WI high signal (**Figures 4a, b**). At the 7-month follow-up, MRI revealed similar findings with persistent striped T1WI low and T2WI high signal (**Figures 4c, d**).

## 3 Discussion

NETs are rare malignancies that originate from neuroendocrine cells and peptide-secreting neurons. While they typically occur in endocrine organs, NETs can also develop in non-endocrine organs and tissues. The most common primary sites are the gastrointestinal tract and lungs, with renal involvement being exceedingly rare (5). Neuroendocrine cells are present in the kidney during fetal development but are generally absent in normal adult kidneys. The pathogenesis of renal NETs remains unclear. The view supported by most scholars is that the original pluripotent stem cells differentiate into neuroendocrine cells (2). However, some scholars have proposed that neural-crest derived cells misplaced or retained within the kidney during embryogenesis may later proliferate or undergo neoplastic transformation, giving rise to tumors with neuroendocrine features (6). Renal carcinoids are associated with certain renal malformations, including horseshoe kidney, mature cystic teratomas, and polycystic kidney disease, with horseshoe kidney being most strongly linked to renal carcinoid. Reports indicate that 18% to 26% of renal carcinoids occur in patients with horseshoe kidney, and these individuals face a 62-fold increased risk of developing NETs (7, 8). Histologically, NETs are classified into well-differentiated types (carcinoid and atypical carcinoid) and poorly differentiated types (small cell carcinoma and large cell carcinoma). According to the 2016 WHO classification of renal tumors, renal endocrine tumors are categorized into four types: paraganglioma, well-differentiated neuroendocrine tumors (carcinoid and atypical carcinoid), small cell neuroendocrine carcinoma, and large cell neuroendocrine carcinoma (9–11).

Clear cell renal cell carcinoma (CCRCC) and NETs share certain biological features: both may exhibit relatively indolent growth yet metastasize at an early stage, and they can display overlapping morphological characteristics. Current research indicates that CCRCC may exhibit neuroendocrine characteristics. Mjønes et al. demonstrated a strong association between clear cell renal cell carcinoma and the expression of erythropoietin and NSE, suggesting that the tumor may originate from erythropoietin-producing cell (EPC). Embryologically, there are many arguments favoring neural crest origin of the erythropoietin-producing cell (12, 13). These findings provide important biological context for the disease and support the consideration of neuroendocrine markers and erythropoietin expression during diagnostic evaluation.

### 3.1 Clinical presentation

PRNETs are most commonly found in individuals aged between 50 and 60 years, predominantly in the right kidney, with no significant gender difference observed (2, 14). Typically, these tumors present with non-specific clinical symptoms and are often discovered incidentally during routine physical exams. When symptoms do appear, patients commonly experience back pain, abdominal distension, abdominal mass, and hematuria (2, 8). Well-differentiated NET cells possess neuroendocrine functionality and can secrete vasoactive substances such as dopamine, histamine, and prostaglandins, which may result in carcinoid syndrome. Studies have shown that approximately 15% of patients with well-differentiated NETs develop carcinoid syndrome, presenting symptoms such as facial flushing, systemic edema, diarrhea, and asthma (10). This type of tumor usually spreads locally and metastasis is rare, but the frequency of metastasis increases with the increase of tumor size (15).

### 3.2 Diagnosis and differential diagnosis

Renal NETs usually present as solid masses with clear boundaries, mostly located near the renal hilum of the renal parenchyma, while those with horseshoe kidney showed cystic-solid masses, and the lesions were mostly located near the isthmus (2). These tumors are generally hypovascular or even avascular, leading to either no enhancement or mild enhancement, with larger tumors occasionally showing heterogeneous enhancement. Approximately 25% of renal NETs exhibit internal calcification, and about 50% of patients have retroperitoneal lymphadenopathy (8, 16, 17). Some studies suggest that calcification is associated with the indolent nature of the tumor, while hemorrhage and necrosis are linked to more aggressive growth and a poorer prognosis (2). Radionuclide-labeled octreotide can specifically bind to somatostatin receptors in tumor tissue, aiding in the detection of primary carcinoid and metastatic lesions. This method has been reported to have a sensitivity greater than 85% for detecting carcinoid tumors, including small and asymptomatic lesions (18). FDG-PET is commonly used to assess tumor metabolism in cancer patients in various stages of the disease (19). In our case, the tumor was located in the left kidney, displaying mild heterogeneous enhancement on contrast-enhanced imaging with calcification, but without retroperitoneal lymphadenopathy. Currently, there is limited imaging data on PRNETs. Based on this case and a review of the literature, the following imaging features can be summarized: heterogeneous density, mild to moderate enhancement, and calcification, which are helpful for diagnosing renal NETs. Most lesions exhibit heterogeneous signal intensity across different sequences, with varying signal characteristics that are not specific and may be related to the presence of cystic degeneration, hemorrhage, or necrosis.

Conventional imaging techniques often struggle to accurately differentiate renal NETs from other renal malignancies, such as renal cell carcinoma, due to overlapping imaging features. This overlap is a key factor contributing to the misdiagnosis in this patient. Clinically, renal NETs must be distinguished from the following conditions: (1) CCRCC: On CT, these tumors typically present as low-density masses with a common pseudocapsule. Microcystic degeneration, hemorrhage, and calcification are often seen. On MRI, clear cell carcinoma usually shows high signal intensity on T2WI, and some lesions may not demonstrate low signal intensity on the ADC map. Post-contrast imaging typically reveals significant enhancement, with a “fast-in, fast-out” enhancement pattern. (2) Chromophobe renal cell carcinoma: These tumors are characterized by a high degree of homogeneity. On CT, they typically show equal density, while MRI often reveals equal signal intensity on T1WI. Enhanced CT and MRI show uniform enhancement, and DWI displays high signal intensity. Delayed imaging may reveal a central strip-like low signal, known as the “spoke-wheel sign” (20, 21). (3) Fat-Poor Renal Angiomyolipoma: These lesions exhibit a typical homogeneous high density on CT and a characteristic homogeneous low signal on T2WI and ADC maps. They lack a capsule, calcification, or hemorrhage. The common enhancement pattern is “fast-in, fast-out,” although approximately one-third of lesions may show delayed enhancement (22, 23).

Although the calcification, mild to moderate enhancement and heterogeneous density in imaging examination are helpful for the diagnosis of well differentiated renal NETs, the final diagnosis and differential diagnosis mainly depend on the results of pathological and immunohistochemical examination. The commonly used neuroendocrine markers are chromogranin A (CgA), neuron-specific enolase (NSE), synaptophysin (Syn) and CD56. Recently, insulinoma-associated 1 (INSM-1), a zinc finger transcription factor, was also found to be expressed in NETs (24). The specificity of CgA in the diagnosis of carcinoid was 97%, while that of Syn was as high as 100% (2). Ki-67 is a reliable marker of pathological grading, and its index reflects cell proliferation activity to a certain extent. The immunohistochemical results of this patient showed that the tumor cells were positive for CK, Syn, CgA, NSE, and INSM-1, and the Ki-67 proliferation index was 2-3%. According to the European Neuroendocrine Tumor Society (ENETS) guidelines and the 2010 World Health Organization (WHO) classification, the NETs of this patient was graded as G1 (25). The final diagnosis was: (left) renal neuroendocrine tumor (NET, G1, carcinoid). Erythropoietin immunohistochemistry could not be performed owing to insufficient residual tissue, a limitation that, although mitigated by the diagnostic support provided by characteristic morphology and standard immunohistochemical markers, should be acknowledged.

### 3.3 Treatment and prognosis

Although primary renal NETs are low-invasive tumors in terms of tumor biology, they still have invasion and metastasis, so they should be treated as soon as possible. Surgery is the best treatment for the disease, and the current gold standard for the treatment of this type of tumor is partial nephrectomy or radical nephrectomy. The extent of surgery mainly depends on the size and location of the tumor, as well as the status of invasion and lymph node metastasis. Radical nephrectomy can be used for large tumors or tumors that cannot be completely resected by partial resection. Lymph node dissection is recommended during surgery, especially if there is any evidence of lymph node enlargement. So far, no systemic therapy has been established for adjuvant or metastatic setting of PRNETs (6, 25, 26). Octreotide is a long-acting somatostatin analogue used for both detection and as a first line antineoplastic systemic therapy. Although radiation therapy has not been extensively studied, radiolabeled octreotide is effective in reducing tumor volume and improving clinical symptoms in patients with metastases (5, 17).

Renal NETs have a lower degree of malignancy and a good prognosis, but long-term follow-up is still needed to monitor the patient’s condition. The following major prognostic factors have been identified. Patients older than 40 years tend to have faster disease progression and more severe initial symptoms. Tumors with a maximum diameter of less than 4 cm or confined to the renal parenchyma usually have fewer metastases and have a better prognosis. Other important prognostic factors include high mitotic rate greater than 2/10 HPF, lymphovascular invasion, atypical cytology and necrosis (17, 26). Given the nonspecific nature of CT and MRI, postoperative imaging should include somatostatin receptor scintigraphy to detect metastatic disease. Long-term follow-up is recommended because metastatic disease can occur even within five years after diagnosis. Follow-up should include physical examination, biochemical laboratory examination and chromogranin A (CgA) levels, along with imaging studies every 3-6 months (6).

## 4 Conclusion

Renal NETs are extremely rare and usually presents as a low-grade malignant tumor with a good prognosis. Due to their similarity to other renal tumors, they are prone to misdiagnosis and delayed diagnosis. Pathological examination and immunohistochemical analysis are essential for accurate diagnosis. Surgical resection is the best treatment modality, and octreotide shows potential as an effective adjuvant therapy for metastatic disease. However, due to the rarity of these tumors, continued research and long-term follow-up are essential to better understand their biological behavior and develop more effective treatment strategies.

## Data Availability

The original contributions presented in the study are included in the article/**Supplementary Material**. Further inquiries can be directed to the corresponding authors.
